# Reliability and Validity of the Australian Mental Health Triage Scale in Children's Hospitals

**DOI:** 10.1111/acem.70426

**Published:** 2026-10-20

**Authors:** Ashley A. Foster, Jacqueline Grupp‐Phelan, Ryman Crone, Marshall Brown, Kim Denicolo, Kate Farley, Elizabeth R. Alpern, Patricia Cobb, Aron Janssen, Jennifer A. Hoffmann

**Affiliations:** ^1^ Department of Emergency Medicine University of California San Francisco California USA; ^2^ Seattle Children's Research Institute Seattle Washington USA; ^3^ Division of Emergency Medicine Ann & Robert H. Lurie Children's Hospital of Chicago Chicago Illinois USA; ^4^ Department of Pediatrics and Medical Social Sciences Northwestern University Feinberg School of Medicine Chicago Illinois USA; ^5^ Pritzker Department of Psychiatry and Behavioral Health Ann & Robert H. Lurie Children's Hospital of Chicago Chicago Illinois USA

## Abstract

**Objective:**

To evaluate interrater reliability and validity of the Australian Mental Health Triage Scale (AMHTS) for children presenting to the emergency department (ED) with behavioral health symptoms.

**Methods:**

We conducted a prospective observational study of ED visits by children aged 3–17 with a primary behavioral health symptom at two US children's hospitals. Nurses assigned Emergency Severity Index (ESI) and AMHTS Scores. Interrater reliability was assessed using intraclass correlation coefficients (ICCs) on visits with two independently assigned AMHTS Scores. To evaluate validity, quasi‐Poisson regression examined the association of AMHTS Scores and ED resource utilization, adjusted for sociodemographic and clinical factors. Resource use was categorized using the standard ESI definition and an expanded definition incorporating behavioral health‐specific resources.

**Results:**

We included 454 visits (45.4% aged 10–14, 56.2% female) with at least one AMHTS Score, of which 94.1% were ESI 2 and 26.9% were AMHTS 2. Among 221 visits with two AMHTS Scores, ICC was 0.48 (95% CI 0.37, 0.58). Higher‐acuity AMHTS Scores were associated with greater resource utilization. Standard resource use was higher for AMHTS Score 1 (Incidence Rate Ratio [IRR] 1.82, 95% CI 1.12, 2.96) and Score 2 (IRR 1.64, 95% CI 1.15, 2.40) versus AMHTS Score 5. When applying the expanded definition, resource use was higher for AMHTS Score 1–4 versus 5.

**Conclusion:**

Interrater reliability of AMHTS was fair, and higher‐acuity scores were associated with greater ED resource utilization. AMHTS Scores were more widely distributed than ESI Scores, suggesting a more responsive approach to pediatric behavioral health triage.

## 1 Introduction

In emergency department (ED) settings, triage serves to identify patients who require immediate attention from those who can safely wait for care [1]. Pediatric EDs across the United States (US) have experienced a marked increase in visits related to behavioral health concerns, inclusive of mental health and substance use disorders [2, 3]. This surge strains already limited resources and contributes to extended wait times for care [4, 5]. Effective triage tools are essential to determine which children presenting with behavioral health symptoms need urgent attention and which can safely wait. The Emergency Severity Index (ESI), a five‐level triage framework, is the most widely implemented triage tool in US EDs [6]. Despite data supporting the reliability and validity of ESI in acute injury and medical contexts [7], emerging evidence suggests important limitations in its performance for pediatric behavioral health presentations, including substantial rates of overtriage and undertriage [8].

Children with behavioral health symptoms are triaged as “high risk” (ESI Score 2) when they present a danger to themselves, others, or the environment [9]. Across EDs, an ESI Score 2 is assigned to most children presenting with behavioral health symptoms [8, 10, 11], which may obscure meaningful differences in acuity within this population. This compression of acuity scores may shift decision‐making toward more subjective clinical assessment, which is prone to the influence of implicit bias [12]. Prior studies have documented racial and ethnic disparities in pediatric ED wait times [12, 13], as well as sociodemographic differences in triage misclassification among children with behavioral health presentations [8]. These limitations underscore the need for structured triage frameworks for pediatric behavioral health presentations that enhance equity, improve classification accuracy, and optimize resource allocation. The Australian Mental Health Triage Scale (AMHTS), broadly used in Australia for behavioral health triage in both adults and children [14], incorporates defined time‐to‐provider targets for each acuity score. In general ED settings, its use has resulted in improved discrimination of urgency and better adherence to evaluation timeframes compared with ESI [15]. However, its performance in pediatric populations has not been well studied. We therefore aimed to prospectively evaluate interrater reliability and validity of the AMHTS as a triage score for children presenting to the ED with behavioral health symptoms.

## 2 Methods

### 2.1 Study Design and Setting

We conducted a two‐site, prospective observational study from December 2024 to September 2025. The two sites were US quaternary children's academic medical centers in differing geographic locations (Midwest, West) with approximately 57,000 and 20,000 visits annually. These results are reported in accordance with the Strengthening the Reporting of Observational Studies in Epidemiology (STROBE) statement [16]. Each site obtained Institutional Review Board approval.

### 2.2 Selection of Participants

We invited ED nurses trained in triage at both sites to participate via email and recruitment flyers.

### 2.3 Study Procedures

Participating nurses completed a 10‐min AMHTS online training module that included a 3‐item self‐assessment. They also completed a one‐time demographic survey which collected information about number of years employed in the current ED, number of years of ED triage experience, gender identity, race, and ethnicity. For each ED visit by a child 3–17 years old with a primary behavioral health symptom (as defined in Table S1), two participating nurses independently completed a standardized data collection form that included an AMHTS reference table, their AMHTS Score assignment, the role of the nurse (triage, bedside, charge, other), and patient identifiers (Figure S1). Nurses were instructed to assign an AMHTS Score within 1 h of patient arrival, and a second nurse was instructed to independently assign an AMHTS Score within 1 h of the first score. AMHTS Scores were only collected for research purposes and were not used for clinical care. Nurse participants received a $5 gift card for completion of each standardized data collection form.

Electronic medical records of included ED visits were retrospectively reviewed. Visit sociodemographic characteristics included age (4–9, 10–14, 15–17), sex (female, male), race and ethnicity (Hispanic, Non‐Hispanic Black, Non‐Hispanic White, Non‐Hispanic other, Non‐Hispanic two or more races), preferred language (English, Spanish, other), insurance (private, government/public, self‐pay/uninsured), and Child Opportunity Index based on home ZIP code (very low, low, moderate, high, very high) [17]. Race, ethnicity, and preferred language were classified as recorded in the electronic health record. Visit characteristics included ED visit season (fall, spring, summer, winter), ED arrival shift (day, evening, night), number of ED patient arrivals on the same calendar day as the ED visit classified into site‐specific tertiles (low, medium, high), ESI Score (1 [highest acuity] to 5 [lowest acuity]), suicide risk screen (Ask Suicide‐Screening Questions response: negative, non‐acute positive, acute positive/imminent risk) [18], mode of arrival (ambulance, other), ED disposition (discharged, admitted to medical unit, admitted or transferred to psychiatric unit, other/unknown), length of ED stay (minutes), presence of aggressive behavior [19], presence of complex chronic condition [20], and return ED visit to the same ED within 30 days. Aggressive behavior and presence of complex chronic condition were determined based on specific discharge diagnoses previously reported in the literature [19, 20].

### 2.4 Outcome Measures

The primary outcome was interrater reliability of AMHTS Score assignment, measured by comparing the assigned AMHTS Score of the two independent nurse raters. Secondary outcomes were: (1) association of AMHTS Score with ED resource utilization, and (2) the proportion of visits with time‐to‐provider evaluation occurring within AMHTS recommended timeframes (AMHTS Score 1 to be seen immediately, AMHTS Score 2 to be seen within 10 min, AMHTS Score 3 to be seen within 30 min, AMHTS Score 4 to be seen within 60 min, AMHTS Score 5 to be seen within 120 min) [21]. We tested two definitions of resource utilization: a standard ESI definition (hereafter, “standard resource definition”), and an expanded definition incorporating behavioral health ED resources (hereafter, “expanded resource definition”). The standard resource definition was based on the ESI Handbook definition of an ED resource, which requires counting the number of anticipated resource types [9]. Each of the following is considered a resource type and assigned 1 point: lab(s), electrocardiogram, radiograph, advanced imaging (e.g., computed tomography scan, magnetic resonance imaging, ultrasound, angiography), intravenous fluids, intravenous or intramuscular or nebulized medication, specialty consultation, and simple procedure [22]. A complex procedure is assigned 2 points [9]. The expanded resource definition incorporating behavioral health resources, developed by the study team, was defined by counting resource types specified in the ESI Handbook as well as the following resource types: mental health specialty consultation, placement in a specified ED safe room, one‐on‐one observation, and physical or pharmacologic restraint administration during the ED visit [23].

### 2.5 Statistical Analysis

We provided characteristics of nurse participants and included ED visits with descriptive statistics and frequency distributions. We assessed interrater reliability among visits with AMHTS Scores assigned by two nurses if the first AMHTS Score was assigned within 1 h of patient arrival and the second AMHTS Score was assigned within 1 h of the first score. Interrater reliability was assessed using intraclass correlation coefficients (ICCs) [24, 25], based on a two‐way random effects model with absolute agreement for the total sample, such that the raters were treated as a random sample. To evaluate whether reliability differed by rater experience, ICCs were additionally calculated within subgroups defined by nurse triage years of experience (both nurses ≤ 7 years, mixed experience, both > 7 years), with the threshold of 7 years chosen to align with prior literature examining AMHTS [26].

We examined the secondary outcomes, ED resource use and time‐to‐provider evaluation among the group of visits with an AMHTS Score assigned by one nurse within 1 h of patient arrival, regardless of the presence or timing of a second AMHTS Score. We determined the associations between ESI Score and ED resource use, as well as AMHTS Score and ED resource use, by using unadjusted and adjusted quasi‐Poisson regression models. Models were adjusted for individual hospital site visit differences, and sociodemographic, clinical, and system‐level factors thought to influence ED resource use: age, sex, race and ethnicity, insurance, mode of arrival, presence of complex chronic condition, number of ED arrivals on the same calendar day, day of the week of arrival, ED arrival shift, and ED visit season. The largest category was used as the reference group unless clinical relevance dictated otherwise. Analyses were conducted using complete case analysis, such that only observations with non‐missing data for all variables included in each model were analyzed. Results were reported as incidence rate ratios (IRR) with 95% confidence intervals (CI). Minutes between ED arrival and provider evaluation were examined by AMHTS Score, and the percentage of visits with provider evaluation occurring within the recommended AMHTS timeframe were quantified. Statistical analysis was conducted with R version 4.5.0 (2025‐04‐11).

## 3 Results

### 3.1 Characteristics of Participating Nurses

There were 69 enrolled nurse participants (Table S2), with 48/57 (84.2%) at site A and 21/45 (46.7%) at site B, and a median of 6 years of experience (interquartile range 2 years, 13 years) conducting ED triage.

### 3.2 Characteristics of Included Emergency Department Visits

Participating nurses assigned AMHTS Scores for 556 ED visits by children presenting with behavioral health symptoms, of which 286 ED visits had an AMHTS Score independently assigned by two nurses (Figure S2). During the study period, 704 visits by children for behavioral health symptoms occurred that did not meet criteria for analysis due to < 1 AMHTS Score determination, AMHTS Score assignment > 1 h after ED arrival, or missing data (Table S3).

After excluding 6 visits due to missing data, 454 ED visits were included in the analytic sample for resource use; of these, 45.4% were by children 10–14 years old, 56.2% were by females, 29.5% were by non‐Hispanic White children, and 37.4% were by Hispanic children (Table 1). Positive suicide risk screen (Ask Suicide‐Screening Questions) was documented in 74.7% of visits, and aggressive behavior in 11.9% of visits. The distribution of ESI Scores was: 94.1% Score 2, 4.8% Score 3, and 1.1% Score 4 (Figure 1). The distribution of AMHTS Scores was: 4.8% Score 1, 26.9% Score 2, 43.4% Score 3, 17.0% Score 4, and 7.9% Score 5. Fifty‐two percent of visits had an evening arrival, with a median length of visit of 361 min (interquartile range 247 min, 543 min; Table 2). A mean of 1.1 resources was utilized (standard deviation 1.1) by standard resource definition criteria, and a mean of 3.6 resources were utilized (standard deviation 1.4) using the expanded resource definition (Figure S3). Eighty‐two percent of ED visits had a mental health consult and 65.9% had at least 1 lab test (Table S4). The majority (61.0%) of visits resulted in discharge.

**TABLE 1:** Sociodemographic characteristics of the study visit cohort.

	Cohort (*N* = 454 [%])
Age (years)	
4–9	67 (14.8)
10–14	206 (45.4)
15–17	181 (39.9)
Sex	
Female	255 (56.2)
Male	199 (43.8)
Race and ethnicity	
Hispanic	170 (37.4)
Non‐Hispanic Black	88 (19.4)
Non‐Hispanic White	134 (29.5)
Non‐Hispanic other	49 (10.8)
Non‐Hispanic two or more races	13 (2.9)
Language	
English	395 (87.0)
Spanish	53 (11.7)
Other	6 (1.3)
Insurance	
Private	215 (47.4)
Government/public	232 (51.1)
Self‐pay/uninsured	7 (1.5)
Childhood Opportunity Index	
Very low	103 (22.7)
Low	22 (4.8)
Moderate	90 (19.8)
High	112 (24.7)
Very high	127 (28.0)

**FIGURE 1:**
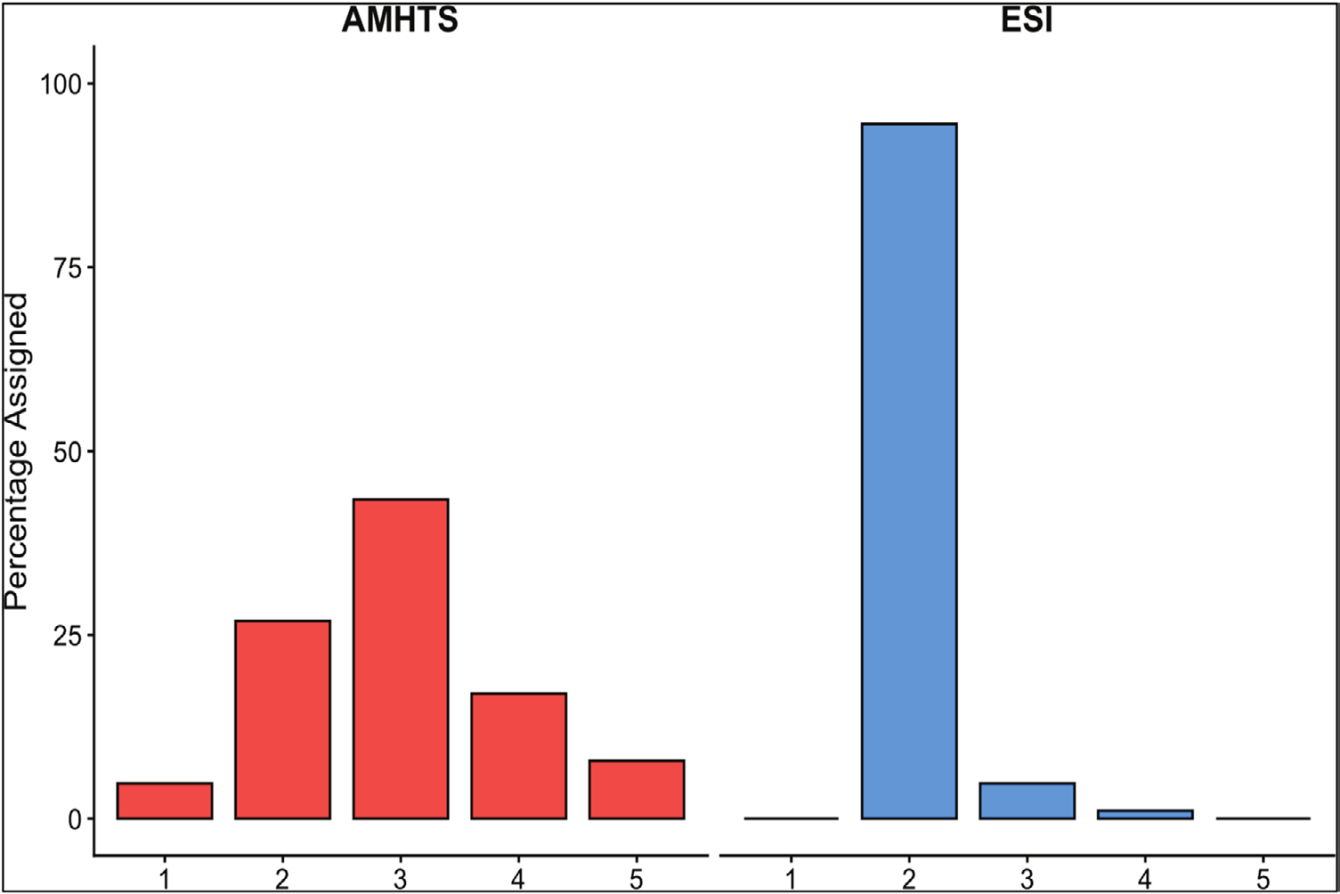
Distribution of acuity scores assigned using the Australian Mental Health Triage Scale (AMHTS) and Emergency Severity Index (ESI). Distribution of percentage of assigned Australian Mental Health Triage Scale and Emergency Severity Index Scores among included pediatric behavioral health emergency department visits.

**TABLE 2:** Clinical characteristics of the study visit cohort.

	Cohort (*N* = 454 [%])
Arrival by ambulance	42 (9.3)
Arrival shift	
Day	193 (42.5)
Evening	240 (52.9)
Night	21 (4.6)
Number of ED patients in 24 h of visit arrival^a^	
Low	145 (31.9)
Medium	153 (33.7)
High	156 (34.4)
Day of week	
Weekday	385 (84.8)
Weekend	69 (15.2)
Season of visit	
Winter	85 (18.7)
Spring	229 (50.4)
Summer	140 (30.8)
Fall	0 (0.0)
Emergency Severity Index Score	
1 (highest acuity)	0 (0.0)
2	427 (94.1)
3	22 (4.8)
4	5 (1.1)
5 (lowest acuity)	0 (0.0)
Australian Mental Health Triage Scale Score	
1 (highest acuity)	22 (4.8)
2	122 (26.9)
3	197 (43.4)
4	77 (17.0)
5 (lowest acuity)	36 (7.9)
Aggressive behavior^b^	54 (11.9)
Complex chronic condition	26 (5.7)
Ask suicide‐screening questions response	
Negative	115 (25.3)
Non‐acute positive	161 (35.5)
Acute positive/imminent risk	178 (39.2)
Disposition	
Discharged	277 (61.0)
Admitted to medical unit	44 (9.7)
Admitted or transferred to psychiatric facility	126 (27.8)
Other/unknown	7 (1.5)
Median length of stay, minutes (interquartile range)	361 (247, 543)
Return ED visit within 30 days	50 (11.0)

^a^ Site‐specific tertiles.

^b^ Unknown *n* = 3.

### 3.3 Interrater Reliability of the Australian Mental Health Triage Scale

Among 221 ED visits in the analytic sample for interrater reliability, the median time between AMHTS Scores was 9 min (interquartile range 3, 22.5 min). The ICC for assigned AMHTS Score was 0.48 (95% CI 0.37, 0.58; Figure S4). The ICC was 0.40 (95% CI 0.15, 0.52) for AMHTS Scores assigned by two nurses with ≤ 7 years of ED triage experience (*n* = 86), 0.50 (95% CI 0.30, 0.65) for two nurses with mixed experience (*n* = 76), and 0.70 (95% CI 0.53, 0.80) for two nurses with > 7 years ED triage experience (*n* = 59).

### 3.4 Association of Assigned Acuity Scores and Emergency Department Resource Use

Unadjusted Poisson regression showed that higher‐acuity ESI Scores were associated with greater resource utilization under both the standard and expanded resource definitions (Table 3). Adjusted Poisson regression revealed that, using the standard resource definition, resource use was significantly greater for ESI 2 (IRR 3.48, 95% CI 1.13, 19.40) compared to ESI 4 (Figure 2). Using the expanded resource definition, resource use was significantly higher for ESI 2 (IRR 6.67, 95% CI 3.33, 16.40) and ESI 3 (IRR 4.16, 95% CI 2.02, 10.40) compared to ESI 4.

**TABLE 3:** Association of Emergency Severity Index (ESI) and Australian Mental Health Triage Scale (AMHTS) Score and resource use.

	Standard resource definition			Expanded resource definition		
	Unadjusted	Adjusted ESI	Adjusted AMHTS	Unadjusted	Adjusted ESI	Adjusted AMHTS
	IRR (95% CI)	IRR (95% CI)	IRR (95% CI)	IRR (95% CI)	IRR (95% CI)	IRR (95% CI)
AMHTS						
1	1.64 (1.00, 2.68)	—	1.82 (1.12, 2.96)	1.85 (1.50, 2.28)	—	1.91 (1.56, 2.34)
2	1.64 (1.14, 2.43)	—	1.64 (1.15, 2.40)	1.71 (1.46, 2.03)	—	1.68 (1.43, 1.97)
3	1.13 (0.79, 1.68)	—	1.08 (0.76, 1.57)	1.53 (1.30, 1.80)	—	1.49 (1.28, 1.74)
4	1.06 (0.70, 1.62)	—	1.03 (0.69, 1.55)	1.27 (1.06, 1.52)	—	1.21 (1.02, 1.44)
5	Reference	—	Reference	Reference	—	Reference
ESI						
2	2.76 (0.88, 17.10)	3.48 (1.13, 19.40)	—	6.09 (2.94, 15.80)	6.67 (3.33, 16.40)	—
3	2.61 (0.76, 16.70)	2.93 (0.88, 16.80)	—	3.94 (1.85, 10.40)	4.16 (2.02, 10.40)	—
4	Reference	Reference	—	Reference	Reference	—
Age (years)						
4–9	0.54 (0.37, 0.75)	0.60 (0.42, 0.83)	0.56 (0.40, 0.77)	0.82 (0.73, 0.92)	0.87 (0.77, 0.97)	0.83 (0.74, 0.93)
10–14	Reference	Reference	Reference	Reference	Reference	Reference
15–17	1.25 (1.04, 1.51)	1.19 (0.99, 1.43)	1.24 (1.04, 1.49)	1.08 (1.00, 1.17)	1.05 (0.98, 1.13)	1.06 (0.99, 1.14)
Sex						
Female	1.14 (0.95, 1.36)	1.12 (0.93, 1.34)	1.12 (0.94, 1.34)	1.04 (0.96, 1.12)	1.05 (0.97, 1.12)	1.04 (0.97, 1.12)
Male	Reference	Reference	Reference	Reference	Reference	Reference
Race and ethnicity						
Hispanic	0.99 (0.80, 1.23)	1.12 (0.88, 1.42)	1.11 (0.88, 1.40)	0.99 (0.91, 1.09)	1.04 (0.95, 1.15)	1.05 (0.95, 1.15)
Non‐Hispanic Black	0.76 (0.57, 0.99)	0.76 (0.57, 1.00)	0.77 (0.58, 1.01)	0.93 (0.84, 1.04)	0.94 (0.84, 1.04)	0.96 (0.87, 1.07)
Non‐Hispanic White	Reference	Reference	Reference	Reference	Reference	Reference
Non‐Hispanic other	1.18 (0.87, 1.57)	1.05 (0.77, 1.42)	1.07 (0.80, 1.43)	1.09 (0.96, 1.24)	1.03 (0.91, 1.17)	1.04 (0.92, 1.17)
Non‐Hispanic two or more races	1.02 (0.58, 1.68)	0.90 (0.51, 1.49)	0.92 (0.53, 1.50)	1.01 (0.80, 1.26)	0.94 (0.76, 1.16)	0.97 (0.78, 1.19)
Language						
English	Reference	Reference	Reference	Reference	Reference	Reference
Spanish	0.98 (0.73, 1.28)	0.78 (0.57, 1.07)	0.77 (0.56, 1.04)	1.02 (0.90, 1.14)	0.97 (0.86, 1.10)	0.96 (0.85, 1.08)
Other	2.16 (1.21, 3.54)	1.64 (0.90, 2.81)	1.65 (0.92, 2.79)	1.37 (1.03, 1.78)	1.19 (0.90, 1.56)	1.18 (0.89, 1.53)
Insurance						
Private	1.07 (0.90, 1.29)	1.09 (0.90, 1.34)	1.07 (0.88, 1.30)	1.08 (1.00, 1.17)	1.08 (0.99, 1.16)	1.09 (1.00, 1.18)
Government/public	Reference	Reference	Reference	Reference	Reference	Reference
Self‐pay/uninsured	0.95 (0.40, 1.87)	0.94 (0.41, 1.87)	0.78 (0.34, 1.55)	0.92 (0.65, 1.24)	1.00 (0.73, 1.34)	0.89 (0.65, 1.19)
Ambulance arrival	1.40 (1.06, 1.82)	1.07 (0.77, 1.49)	0.98 (0.70, 1.35)	1.15 (1.02, 1.30)	1.08 (0.94, 1.24)	1.07 (0.93, 1.23)
Chronic complex condition	1.71 (1.26, 2.29)	1.73 (1.26, 2.32)	1.65 (1.22, 2.19)	1.19 (1.02, 1.37)	1.20 (1.05, 1.38)	1.15 (1.01, 1.32)
Shift arrival						
Day	1.01 (0.84, 1.21)	1.03 (0.86, 1.24)	1.06 (0.88, 1.26)	1.02 (0.95, 1.10)	1.04 (0.97, 1.11)	1.06 (0.99, 1.14)
Evening	Reference	Reference	Reference	Reference	Reference	Reference
Night	1.05 (0.67, 1.57)	1.06 (0.68, 1.57)	1.01 (0.66, 1.49)	1.01 (0.84, 1.21)	1.06 (0.90, 1.25)	1.07 (0.91, 1.26)
Day of the week						
Weekday	Reference	Reference	Reference	Reference	Reference	Reference
Weekend	1.04 (0.81, 1.32)	1.12 (0.88, 1.43)	1.06 (0.83, 1.35)	0.98 (0.89, 1.09)	1.01 (0.91, 1.11)	0.98 (0.89, 1.08)
Visit season						
Spring	Reference	Reference	Reference	Reference	Reference	Reference
Summer	1.21 (0.99, 1.47)	1.19 (0.95, 1.49)	1.13 (0.91, 1.40)	1.06 (0.98, 1.15)	1.06 (0.97, 1.16)	1.00 (0.91, 1.09)
Winter	1.02 (0.80, 1.31)	0.92 (0.72, 1.18)	0.88 (0.69, 1.12)	1.00 (0.90, 1.10)	0.96 (0.87, 1.05)	0.94 (0.85, 1.03)
Number of ED patients checked in on assessment day						
Low	1.02 (0.82, 1.27)	0.93 (0.73, 1.17)	0.97 (0.77, 1.22)	1.04 (0.95, 1.14)	0.99 (0.90, 1.09)	1.05 (0.95, 1.15)
Medium	Reference	Reference	Reference	Reference	Reference	Reference
High	0.93 (0.75, 1.16)	1.00 (0.81, 1.24)	1.03 (0.83, 1.27)	1.02 (0.93, 1.12)	1.04 (0.95, 1.13)	1.05 (0.97, 1.15)

**FIGURE 2:**
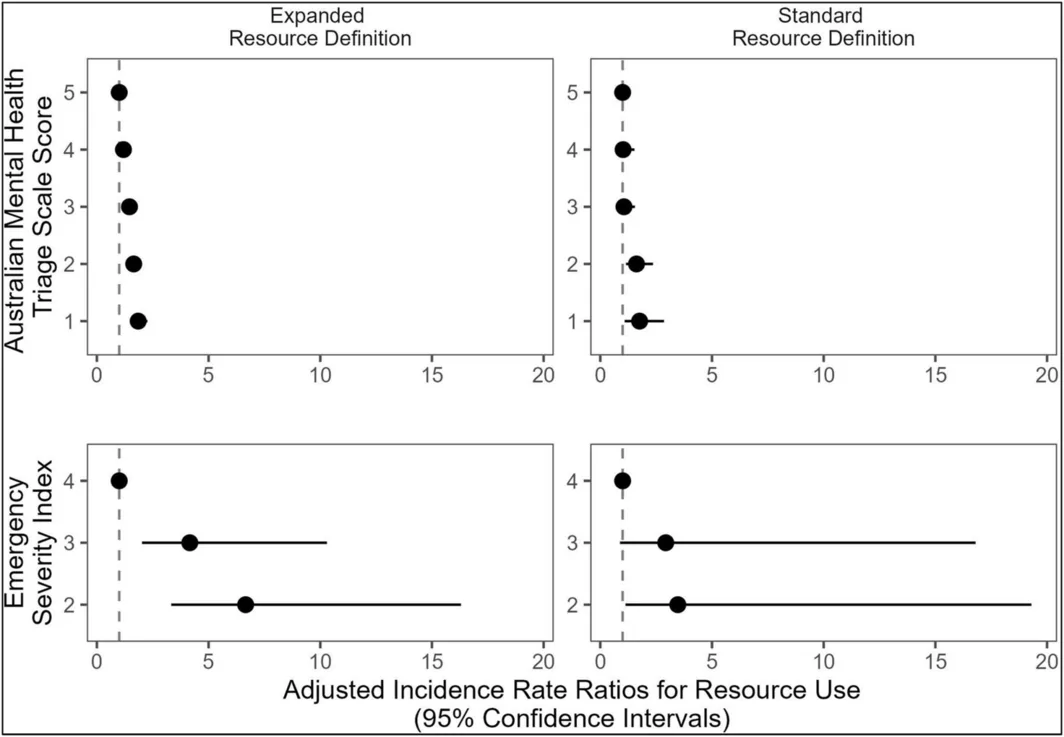
Association of Australian Mental Health Triage Scale Score and Emergency Severity Index and resource use. Forest plots of adjusted quasi‐Poisson regression models evaluating the association between Australian Mental Health Triage Scale and emergency department resource utilization and the association of Emergency Severity Index and emergency department resource utilization.

In unadjusted Poisson regression models, higher‐acuity AMHTS Scores were associated with increased resource utilization under both standard and expanded resource definitions. In adjusted analysis, using the standard resource definition, resource use was significantly greater for AMHTS 1 (IRR 1.82, 95% CI 1.12, 2.96) and AMHTS 2 (IRR 1.64, 95% CI 1.15, 2.40) relative to AMHTS 5. Using the expanded resource definition, resource use was significantly higher for AMHTS 1–4 compared to 5.

### 3.5 Percentage of Visits Meeting Recommended Time‐to‐Provider Timeframe

The median minutes between ED arrival and provider evaluation was 25 min (interquartile range 14 min, 53 min; Table S5). Two hundred and seventeen visits (47.8%) met the AMHTS‐recommended time‐to‐provider evaluation timeframe, with 0% meeting the timeframe for visits with an AMHTS Score of 1 and 97.1% for an AMHTS Score of 5.

## 4 Discussion

In this prospective observational study conducted among triage nurses in two pediatric EDs, we identified fair interrater reliability of AMHTS Scores applied for behavioral health presentations, and we found that AMHTS Score assignment was associated with the number of resources used during the ED visit in a stepwise fashion. Over 90% of included visits were assigned an ESI Score of 2, while AMHTS Scores were more broadly distributed, with roughly 2 in 5 visits assigned an AMHTS Score of 3. Interrater reliability for AMHTS was fair among nurse pairs with fewer years of triage experience, while interrater reliability was good among nurse pairs with greater triage experience. Alignment of assigned AMHTS Scores with resources was robust to different definitions of ED resource use, including the standard ESI resource definition and an expanded definition that encompassed behavioral health‐specific resources. Despite a median 25‐min time‐to‐provider evaluation, fewer than half of visits met AMHTS‐recommended timeframes.

This study's findings indicate fair interrater reliability for AMHTS overall with substantially higher interrater reliability among nurses with more than 7 years of ED triage experience. This pattern suggests that consistent application of AMHTS in usual‐care settings may depend on experiential familiarity with triage and ED workflows, or with nuanced presentations of pediatric behavioral health crises, consistent with prior literature of other scoring systems [27, 28]. In contrast, Hoffmann et al. reported that interrater reliability in pediatric ED behavioral health triage using AMHTS did not meaningfully differ by years of nurse experience, when evaluated using standardized written case scenarios rather than patient encounters in clinical practice [26]. Differences in these findings may reflect the contexts in which reliability was assessed: simulated cases may provide a more uniform clinical frame of reference for all raters, whereas patient encounters in clinical practice may introduce greater variability in presentations and added cognitive load due to competing demands, influencing clinical assessments. In addition to experience, variability in AMHTS scoring among presentations encountered in clinical practice may also reflect patient‐, provider‐, and system‐level factors, including communication barriers, workflow pressures (e.g., ED crowding, interpreter availability), and differences in provider comfort with behavioral health presentations. These factors represent important targets for future investigation to better understand drivers of variability of AMHTS scoring. Nurses in both studies completed a 10‐min training on the behavioral health triage tools; it is possible that in‐person didactics or longitudinal training by experienced triage nurses may be necessary to achieve consistent scoring across all experience levels in live clinical settings [29].

In the ED setting, triage systems are designed to rapidly categorize patients according to anticipated care intensity [9]. In this study, higher acuity scores under both ESI and AMHTS were associated with greater resource utilization, supporting their validity for behavioral health presentations. However, the distribution and strength of these associations differed. The large majority of ESI 2 assignments may limit nuanced differentiation of behavioral health acuity using ESI. In contrast, AMHTS distributed visits across several middle‐acuity scores and demonstrated a more graded relationship with subsequent resource utilization. Greater differentiation within behavioral triage categories may support more structured and consistent decision‐making by triage nurses, rather than reliance on subjective assessment, which is susceptible to implicit bias. Prior study of US pediatric behavioral health ED visits revealed increased adjusted odds of overtriage or undertriage among non‐Hispanic Black children compared to non‐Hispanic White children and children with preferred Spanish language compared to English [8]. By distributing patients more evenly across acuity scores, AMHTS may have the potential to reduce sociodemographic triage disparities related to subjective scoring. Evaluation of AMHTS performance across demographically diverse populations will be essential following implementation.

Given the widespread adoption of ESI in US EDs [6], integration of structured behavioral elements within the pre‐existing ESI framework may offer a pragmatic approach to improving differentiation within behavioral health presentations while preserving familiarity for frontline staff. This approach has been evaluated in a pediatric ED with integrated behavioral health staff, enhanced staff training, and separate spaces for high‐ and low‐acuity patients. Implementation in this setting was associated with decreased staff injury, fewer security codes, and reductions in length of stay and admission rates [30]. Future research should evaluate the feasibility of implementing this approach in routine ED practice, particularly in settings with varying levels of behavioral health expertise and resources. In addition, its effects on triage time, training requirements, and interrater agreement among providers should be assessed. Moreover, multi‐site ED implementation studies could further determine whether embedding behavioral‐specific criteria within established triage processes improves the consistency and usability of behavioral health acuity classification.

## 5 Limitations

This study has several limitations. First, as a two‐site study, findings may not be generalizable to EDs with different patient populations, staffing models, or mental health resources. Second, selection bias may have occurred if characteristics of nurse participants differed from non‐participants, or if eligible encounters were missed due to nurse availability or operational constraints. Third, infrequent occurrence of patient presentations with very high and very low acuity may have reduced precision estimates for these groups. Fourth, ED system constraints, including crowding, staffing, and behavioral health resource availability may have influenced resource utilization and throughput (e.g., time‐to‐provider) independent of triage score, resulting in unmeasured confounders. Lastly, return ED visits may have occurred to non‐study EDs that were not reflected in available data.

## 6 Conclusions

In this prospective study of pediatric behavioral health triage, AMHTS demonstrated fair interrater reliability overall, with substantial agreement among more experienced triage nurses, supporting its reproducibility in clinical practice. Both AMHTS and ESI showed meaningful associations between higher acuity and greater ED resource utilization, reinforcing their alignment with operational ED care intensity. However, the broader distribution of AMHTS Scores compared with the concentration of visits within an ESI Score of 2 suggests that behavioral health severity may be more heterogeneous than reflected within existing triage categorization. These findings support further study of AMHTS both as a behavioral health‐specific triage tool and as a potential component of future refinements to established ED triage frameworks. Future research should evaluate whether training and prospective implementation of AMHTS improves triage accuracy and promotes equitable care for children presenting with behavioral health symptoms.

## Author Contributions

Ashley A. Foster and Jennifer A. Hoffmann conceptualized and designed the study, coordinated and supervised data collection and interpretation, drafted the initial manuscript, and reviewed and revised the manuscript. Marshall Brown carried out data analysis and interpretation and reviewed and revised the manuscript. Ryman Crone, Kim Denicolo, Kate Farley, Elizabeth R. Alpern, Patricia Cobb, Aron Janssen, and Jacqueline Grupp‐Phelan interpreted the data and critically reviewed and revised the manuscript. All authors have approved the final manuscript as submitted and agree to be accountable for all aspects of the work.

## Funding

This study was funded by the Pediatric Pandemic Network. The Pediatric Pandemic Network is supported in part by the Health Resources and Services Administration (HRSA) of the U.S. Department of Health and Human Services (HHS) as part of cooperative agreements U1IMC43532 and U1IMC45814 with 0% financed with nongovernmental sources. The content presented here is that of the authors and does not necessarily represent the official views of, nor an endorsement by HRSA, HHS, or the U.S. Government. For more information, visit HRSA.gov. Research reported in this publication was supported by the National Institutes of Mental Health of the National Institutes of Health under Award Number K23MH135206–01 (to JAH). The content is solely the responsibility of the authors and does not necessarily represent the official views of the National Institutes of Health. Dr. Foster receives funding from Abbott Laboratories and CARESTAR Foundation for studies outside of the submitted work.

This manuscript is the result of funding in whole or in part by the NIH. It is subject to the NIH Public Access Policy. Through acceptance of this federal funding, NIH has been given the right to make this manuscript publicly available in PubMed Central upon the Official Date of Publication, as defined by NIH.

## Conflicts of Interest

The authors declare no conflicts of interest.

## Supporting information


**Figure S1:** Standardized data collection form.
**Figure S2:** Emergency department visits included and excluded from the study cohort.
**Figure S3:** Distribution of resources used during ED visits by standard and expanded resource definitions.
**Figure S4:** Heat map of Australian Mental Health Triage Scale Scores 1 and 2 for each emergency department visit.
**Table S1:** Behavioral chief complaints.
**Table S2:** Nurse participant characteristics.
**Table S3:** Characteristics of ED visits by children < 18 years old with a behavioral health chief complaint during the study period, included and excluded from analysis.
**Table S4:** Resource utilization among the study ED visit cohort.
**Table S5:** Time‐to‐provider for study emergency department visit cohort.

## Data Availability

The data that support the findings of this study are available on request from the corresponding author. The data are not publicly available due to privacy or ethical restrictions.
