# Supplementary material for: Reliability and Validity of the Australian Mental Health Triage Scale in Children's Hospitals
Source: Acad Emerg Med. 2026 Oct 20;33(10):e70426. doi: 10.1111/acem.70426 (PMC13682278; doi:10.1111/acem.70426)
Supplement: Supplementary file 1 [file ACEM-33-0-s001.docx]

**Supplemental Material**

**Table S1.** Behavioral Chief Complaints

**Table S2.** Nurse Participant Characteristics

**Table S3.** Characteristics of ED Visits by Children <18 Years Old with a Behavioral Health Chief Complaint During the Study Period, Included and Excluded from Analysis

**Table S4.** Resource Utilization Among the Study ED Visit Cohort

**Table S5.** Time-to-Provider for Study Emergency Department Visit Cohort

**Figure S1.** Standardized Data Collection Form

**Figure S2.** Emergency Department Visits Included and Excluded from the Study Cohort

**Figure S3.** Distribution of Resources Used During ED Visits by Standard and Expanded Resource Definitions

**Figure S4.** Heat Map of Australian Mental Health Triage Scale Scores 1 and 2 for Each Emergency Department Visit

**Table S1. Behavioral Chief Complaints**

| **Site A** | **Site B*** |
| --- | --- |
| Behavior Concern | Anxiety-Related (e.g., anxiety, panic attack, hyperventilation) |
| Behavior Problem | Substance-Related (e.g., alcohol intoxication, drug overdose, intentional ingestion) |
| Behavioral Health Signs and/or Symptoms | Depression or Self-Harm (e.g., depression, self-injury, suicidal, suicide attempt) |
| Psychological Evaluation | Aggression or Harm to Others (e.g., aggressive behavior, agitation, behavioral concerns or behavioral problem, violent or threatening behavior) |
| Ingestion (if ≥10 years) | Disordered Eating (e.g., anorexia, eating concerns weight loss, eating disorder) |
|  | General Psychiatric/Mental Health Concern |

*****For site B, every chief complaint was reviewed by the first and second author, with agreement required for classification as a primary behavioral health symptom.

**Table S2. Nurse Participant Characteristics**

|  | **Overall**  **N = 69***^1^* | **Site A**  **N=48***^1^* | | **Site B**  **N=21***^1^* | | **p-value***^2^* | |
| --- | --- | --- | --- | --- | --- | --- | --- |
| **Gender** |  |  | |  | |  | |
| Male | 7 (10.1) | 5 (10.4) | | 2 (9.5) | | >0.9 | |
| Female | 61 (88.4) | 43 (90.0) | | 18 (85.7) | | 0.7 | |
| Not Disclosed | 0 (0.0) | 0 (0.0) | | 1 (4.8) | |  | |
| **Race** |  |  | |  | | <0.001 | |
| Asian | 4 (5.9) | 0 (0.0) | | 4 (19.0) | |  | |
| Black or African  American | 2 (2.9) | 0 (0.0) | | 2 (9.5) | |  | |
| White | 59 (86.8) | 46 (95.8) | | 13 (61.9) | |  | |
| More than one race | 3 (4.4) | 1 (2.1) | | 2 (9.5) | |  | |
| Unknown | 1 (1.4) | 1 (2.1) | | 0 (0.0) | |  | |
| **Ethnicity** |  |  | |  | | >0.9 | |
| Hispanic | 6 (9.0) | 4 (8.3) | | 2 (9.5) | |  | |
| Non-Hispanic | 61 (91.0) | 44 (91.7) | | 17 (81.0) | |  | |
| Unknown | 2 (2.9) | 0 (0.0) | | 2 (9.5) | |  | |
| **Years of experience working at current emergency department** | 4 (2, 9) | 3 (2,8) | | 7 (3,10) | | 0.3 | |
| **Years of experience working in emergency department triage** | 6 (2, 13) | 4 (2,8) | | 10 (7,15) | | 0.008 | |
| *^1^*n (%); Median (Q1, Q3) | | |  | |  | |  |

**Table S3. Characteristics of ED Visits by Children <18 Years Old with a Behavioral Health Chief Complaint During the Study Period, Included and Excluded from Analysis**

|  | **Visits with ≥1 AMHTS assigned within 1 hour of arrival and no missing ED visit data** | | |
| --- | --- | --- | --- |
|  | Yes | No | p-value |
|  | N=454  N (%) | N=704  N (%) |  |
| **Age (years)** |  |  | 0.014 |
| 3-9 | 67 (14.8) | 78 (11.1) |  |
| 10-14 | 206 (45.4) | 287 (40.8) |  |
| 15-17 | 181 (39.9) | 339 (48.2) |  |
| **Sex** |  |  | 0.600 |
| Female | 255 (56.2) | 385 (54.7) |  |
| Male | 199 (43.8) | 318 (45.2) |  |
| **Race** |  |  | 0.200 |
| Hispanic | 170 (37.4) | 251 (35.7) |  |
| Non-Hispanic Black | 88 (19.4) | 138 (19.6) |  |
| Non-Hispanic White | 134 (29.5) | 190 (27.0) |  |
| Non-Hispanic other | 49 (10.8) | 87 (12.4) |  |
| Non-Hispanic two or more races | 13 (2.9) | 38 (5.4) |  |
| **ESI Score** |  |  | <0.001 |
| 1 | 0 (0.0) | 3 (0.4) |  |
| 2 | 427 (94.5) | 547 (77.7) |  |
| 3 | 22 (4.8) | 122 (17.3) |  |
| 4 | 5 (1.1) | 31 (4.4) |  |
| 5 | 0 (0.0) | 1 (0.1) |  |
| **Disposition** |  |  | <0.001 |
| Discharged | 277 (61.0) | 394 (56.0) |  |
| Admitted to Medical Unit | 44 (9.7) | 98 (13.9) |  |
| Admitted or Transferred to Psychiatric  Facility | 126 (27.8) | 171 (24.3) |  |
| Other/Unknown | 7 (1.5) | 41 (5.8) |  |

**Table S4. Resource Utilization Among the Study ED Visit Cohort**

|  | N (%)  N = 454 |
| --- | --- |
|  |  |
| Placement in a safe emergency department room | 401 (88.3) |
| Mental health consult | 373 (82.2) |
| Observer assigned | 318 (70.0) |
| Labs | 299 (65.9) |
| Electrocardiogram performed | 63 (13.9) |
| Consult from a specialty other than mental health | 45 (9.9) |
| Intramuscular medication administered | 23 (5.1) |
| Intravenous fluids administered | 22 (4.8) |
| Pharmacologic restraint | 20 (4.4) |
| Radiograph performed | 16 (3.5) |
| Intravenous medication administered | 13 (2.9) |
| Advanced imaging performed | 7 (1.5) |
| Simple procedure performed | 6 (1.3) |
| Order placed for physical restraints | 3 (0.7) |
| Nebulized medication administered | 2 (0.4) |
| Complex procedure performed | 1 (0.2) |

**Table S5. Time-to-Provider for Study Emergency Department Visit Cohort**

|  | | **Australian Mental Health Triage Scale Score** | | | | |
| --- | --- | --- | --- | --- | --- | --- |
|  | **Overall**  **N= 452** | **1**  **N=22** | **2**  **N=122** | **3**  **N=196** | **4**  **N=77** | **5**  **N=35** |
| **Time (minutes) between emergency department triage and provider evaluation**  Median,  (Interquartile range) | 25 (14,53) | 22 (11,28) | 21 (13,45) | 30 (19,64) | 25 (14,56) | 25 (14,52) |
| **Seen within recommended timeframe*** (N, %) | 217 (47.8) | 0 (0.0) | 21 (17.2) | 101 (51.5) | 61 (79.2) | 34 (97.1) |

**Australian Mental Health Triage Scale recommended time to provider evaluation based on score- 1=immediately, 2=within 10 minutes, 3= within 30 minutes, 4= within 60 minutes, 5= within 120 minutes*

**Figure S1. Standardized Data Collection Form**

**Figure S2. Emergency Department Visits Included and Excluded from the Study Cohort**

*Missingness for the following variables: race and ethnicity

**Figure S3. Distribution of Resources Used During ED Visits by Standard and Expanded Resource Definitions**

**Figure S4. Heat Map of Australian Mental Health Triage Scale (AMHTS) Scores 1 and 2 for Each Emergency Department Visit**
